# Online Interventions for Social Marketing Health Behavior Change Campaigns: A Meta-Analysis of Psychological Architectures and Adherence Factors

**DOI:** 10.2196/jmir.1367

**Published:** 2011-02-14

**Authors:** Brian Cugelman, Mike Thelwall, Phil Dawes

**Affiliations:** ^1^Statistical Cybermetrics Research GroupWolverhampton Business SchoolUniversity of WolverhamptonWolverhamptonUnited Kingdom

**Keywords:** Meta-analysis, intervention studies, behavioral medicine, social marketing, behavior, psychology, motivation, online systems, Internet, Web-based services

## Abstract

**Background:**

Researchers and practitioners have developed numerous online interventions that encourage people to reduce their drinking, increase their exercise, and better manage their weight. Motivations to develop eHealth interventions may be driven by the Internet’s reach, interactivity, cost-effectiveness, and studies that show online interventions work. However, when designing online interventions suitable for public campaigns, there are few evidence-based guidelines, taxonomies are difficult to apply, many studies lack impact data, and prior meta-analyses are not applicable to large-scale public campaigns targeting voluntary behavioral change.

**Objectives:**

This meta-analysis assessed online intervention design features in order to inform the development of online campaigns, such as those employed by social marketers, that seek to encourage voluntary health behavior change. A further objective was to increase understanding of the relationships between intervention adherence, study adherence, and behavioral outcomes.

**Methods:**

Drawing on systematic review methods, a combination of 84 query terms were used in 5 bibliographic databases with additional gray literature searches. This resulted in 1271 abstracts and papers; 31 met the inclusion criteria. In total, 29 papers describing 30 interventions were included in the primary meta-analysis, with the 2 additional studies qualifying for the adherence analysis. Using a random effects model, the first analysis estimated the overall effect size, including groupings by control conditions and time factors. The second analysis assessed the impacts of psychological design features that were coded with taxonomies from evidence-based behavioral medicine, persuasive technology, and other behavioral influence fields. These separate systems were integrated into a coding framework model called the communication-based influence components model. Finally, the third analysis assessed the relationships between intervention adherence and behavioral outcomes.

**Results:**

The overall impact of online interventions across all studies was small but statistically significant (standardized mean difference effect size d = 0.19, 95% confidence interval [CI] = 0.11 - 0.28, *P* < .001, number of interventions k = 30). The largest impact with a moderate level of efficacy was exerted from online interventions when compared with waitlists and placebos (d = 0.28, 95% CI = 0.17 - 0.39, *P* < .001, k = 18), followed by comparison with lower-tech online interventions (d = 0.16, 95% CI = 0.00 - 0.32, *P* = .04, k = 8); no significant difference was found when compared with sophisticated print interventions (d = –0.11, 95% CI = –0.34 to 0.12, *P* = .35, k = 4), though online interventions offer a small effect with the advantage of lower costs and larger reach. Time proved to be a critical factor, with shorter interventions generally achieving larger impacts and greater adherence. For psychological design, most interventions drew from the transtheoretical approach and were goal orientated, deploying numerous influence components aimed at showing users the consequences of their behavior, assisting them in reaching goals, and providing normative pressure. Inconclusive results suggest a relationship between the number of influence components and intervention efficacy. Despite one contradictory correlation, the evidence suggests that study adherence, intervention adherence, and behavioral outcomes are correlated.

**Conclusions:**

These findings demonstrate that online interventions have the capacity to influence voluntary behaviors, such as those routinely targeted by social marketing campaigns. Given the high reach and low cost of online technologies, the stage may be set for increased public health campaigns that blend interpersonal online systems with mass-media outreach. Such a combination of approaches could help individuals achieve personal goals that, at an individual level, help citizens improve the quality of their lives and at a state level, contribute to healthier societies.

## Introduction

Research suggests that online intervention can motivate people to adopt healthy behaviors, such as reducing binge drinking [1], stopping smoking [2], and managing healthy weight [3]. Frequently, these online interventions are individually tailored programs, resembling two-way interpersonal therapy. It is now conceivable that health campaigners can deploy mass-interpersonal campaigns, where online media are used to engage large populations in automated relationships that resemble the support offered by dieticians, fitness trainers, or smoking cessation counselors.

At present, numerous factors are driving health promotion campaigns online. First, the Internet offers health campaigners a convenient channel to increase the reach of large-scale campaigns. The Internet is now a major source of information for health advice [4], and presently there are over 1.5 billion Internet users [5]. Second, interactivity offers many benefits and may render online communication more effective than traditional approaches [6-8]. In this regard, online communications can utilize multimedia and interactive capabilities, which offer new ways to engage public audiences. Third, meta-analyses demonstrate that online interventions can match and occasionally outperform traditional interventions [8-10]. Systematic reviews tend to be less conclusive but still show a marginal advantage over traditional interventions [11,12].

Fourth, the cost-effectiveness of preventative medicine and online outreach are both driving the innovation of online health solutions. Governments are recognizing that it is more cost-effective to market healthy lifestyles rather than pay to treat the outcomes of unhealthy lifestyles [13]. This is set against a backdrop where rising health care costs are driving the search for affordable eHealth solutions [14]. Some preventative lifestyle programs have offered significant costs savings to insurance companies in the range of 50% within one year and 20% to 30% in subsequent years [15]. Given the reach and interactivity of the Internet, transcribing these programs to online contexts can bring these types of lifestyle programs to millions but at a fraction of the cost of traditional interventions. For instance, smoking cessation telecounseling interventions were estimated to cost US $150 to US $250 per smoker, tailored print interventions ranged from US $5 to US $40 per smoker, while tailored online smoking cessation interventions could cost less than US $1 per smoker, depending on the population size [2].

When designing campaigns to enhance citizen well-being, health officials draw from numerous fields, theories, frameworks, and techniques. With almost 40 years of academic and practical development, social marketing is an established approach to behavioral change [16]. Social marketing is the use of marketing principles and techniques to influence a target audience to voluntarily accept, reject, modify, or abandon a behavior for the benefit of individuals, groups, or society as a whole [17]. It is based on influencing voluntary behavior, often through incentives in the form of marketing offers targeted to key population segments [18]. It is commonly used by governmental health departments—such as Health Canada [19], the United Kingdom’s Department of Health [20], and the United States’ Centers for Disease Control and Prevention [21]—to design large-scale campaigns promoting healthy lifestyles to millions.

### Designing Online Behavioral Change Interventions 

Social marketers frequently use the Internet to promote healthy lifestyles as part of multichannel campaigns, increasingly with social media tools. However, several authors have argued that new media have introduced changes that are shifting how social marketing campaigns should be carried out and that the old one-way communication model does not make sense in online environments [22] or that social marketers have not yet taken full advantage of the Internet’s potential [23]. These criticisms may be due to the lack of empirical research that can inform the design of online interventions suitable to social marketing contexts.

To understand how online intervention design can influence users' behaviors, some researchers have examined health behavioral change interventions that can be found through Internet search engines. Their studies tend to offer uncertain and sometimes pessimistic conclusions. One evaluation of existing health behavioral change websites concluded that many of these sites did not include the basic requirements to achieve health behavior change [24]. Another study of physical activity websites assessed the extent to which interventions appeared to reflect various behavioral change theories and techniques. The authors concluded that interventions provided little assessment, feedback, or tailored support. Given the lack of intervention features believed to influence behavior, the authors called for more randomized controlled trials to assess long-term impacts [25]. Another research team concluded that government anti-tobacco websites lacked the capacity to disseminate persuasive communications, while grassroots organizations offered the only viable online outreach due to their advocacy capacity [26]. A similar class of research are case studies of online campaigns [27-30]. They often provide in-depth descriptions of particular campaigns and their associated online interventions. These studies provide useful details on how applied online interventions are designed, and they also make the case for how interventions should be designed, but they do not offer empirical evidence that online intervention design is associated with behavioral impacts.

Other types of research that can inform intervention design include meta-analyses [8-10] and systematic reviews [12,31] of online interventions. These studies suggest that online interventions offer small advantages over traditional intervention media, such as websites versus print publications. In some cases, these studies provide insights into intervention design features associated with behavioral impacts. However, these prior studies are limited in their ability to generalize to numerous campaign contexts, where large-scale social marketing campaigns routinely focus on voluntary behavioral change. This is because these prior review studies have not distinguished between interventions targeting behaviors that are voluntary and those that are mandatory. Rather, these studies have pooled interventions targeting voluntary behaviors more suitable to social marketing applications, along with mandatory behaviors that are more suitable to medical applications, such as managing chronic diseases or coping with psychological disorders. Perhaps one exception was a systematic review that offered good evidence that online interventions can influence voluntary behaviors but lacked the statistical insight offered by meta-analysis [11].

Thus far, no meta-analyses have quantified how the psychological design of online interventions can influence behaviors that are typically targeted in social marketing campaigns. To overcome this gap, there is a need to identify a sample of online behavioral change interventions that resembles those used in large-scale public health campaigns and which also offers insight into the psychological architectures associated with voluntary behavioral change.

### Dose

In clinical studies, the more people adhere to lifestyle change programs, the more their health improves. Similarly, those with life threatening diseases who stick to diet and lifestyle programs can potentially prevent their condition from worsening [15]. However, in longitudinal studies of interventions that are neither mandatory nor critical to participants’ well-being, one can expect significant attrition [32]. This trend has prompted researchers to focus on strategies to increase adherence to online interventions [33].

Research suggests that exposure to programs (their dose), is a key predictor of behavior change. In one systematic review, the majority of participants failed to engage in more than half of the expected eHealth activities. However, those interventions with high utilization showed better behavioral outcomes [11]. Similarly, high attrition in person-to-person health behavioral change programs has prompted researchers to argue that online interventions need to put in more effort to prevent dropouts in person-to-computer interventions [24, 32].

In this paper, the term *attrition* describes the proportion of people who stop using an intervention over time [32]. The opposite of this term is *adherence,* which describes the proportion of participants who continue using an intervention over time. Regardless of which term is used, the amount of exposure that people receive when using an intervention is also called *dose*. For interventions that are not mandatory, and participation is voluntary, users will receive a dose that is proportional to their chosen level of adherence or attrition.

There are two types of adherence. First, *intervention adherence* describes the proportion of participants who use an intervention over time. This is negatively called *nonusage attrition* [32]. Second, *study adherence* describes the proportion of participants who stay in a study over time. It is negatively called *dropout attrition* [32], which describes participants who leave a study. Under the law of attrition, it has been proposed that study adherence and intervention adherence are correlated and explained in part by a third variable: *participant interest*, which is in turn influences by other factors, such as usability, push factors, personal contact, positive feedback, peer-to-peer communication, etc. [32]. As intervention adherence is considered critical to intervention efficacy, and study adherence and intervention adherence are believed to be related, there is a need to empirically investigate these relationships.

### Describing the Design of Online Interventions

Although online interventions are frequently described as a homogenous group, they may be radically different in terms of their purpose, design, and psychological architectures. In order to describe the diversity of existing online interventions, any coding system would need to accommodate a large variety of complex factors that may explain intervention efficacy. However, there is no consensus on what constitutes the best theoretical framework or list of factors that may be used to describe interventions and which may also explain their efficacy. The literature offers numerous competing behavioral change theories and taxonomies that are founded on different assumptions, application contexts, and academic disciplines. This has resulted in numerous overlapping and ill-fitting taxonomies, none of which is comprehensive enough to describe online interventions on their own [34, 35]. Moreover, the majority of online health intervention design guidelines do not focus on behavioral outcomes, which renders them inappropriate for assessing design factors that may be associated with behavioral outcomes. For instance, one review of 20 health intervention guidelines found that just 2 addressed outcomes [36].

To overcome the lack of intervention design guidelines addressing behavioral outcomes, this study first reviewed numerous influence systems and then developed a communication-based framework to consolidate taxonomies across various fields into a simple coding system. When describing these various systems, the following terms are used: *influence system* describes any research that classifies approaches to psychological and/or behavior change, and *influence component* describes a particular technique or package of techniques designed to influence a person’s psychology and/or behavior. The review looked at influence systems from evidence-based behavioral medicine [37-40], persuasive technology and the media equation [7,41,42], persuasive communication research [43-45], stages of change [46,47], and community-based social marketing [48,49]. Of these various systems, two influence system taxonomies offered highly robust coding guidelines that reflected commonly reported behavioral change techniques and psychological constructs [38,50]. However, across all studies, no single system was suitable to coding online intervention psychology on their own.

In order to develop a comprehensive coding system to describe the psychological architectures of online interventions, a model was developed to consolidate influence systems across a range of fields. It is called the communication-based influence components model (CBICM). The model views interaction between audiences and online interventions as roughly equal to the relationship between a therapist and client, where the therapist’s treatment is just one of many factors that may explain efficacy. For instance, many therapists may offer the same treatment to their patients; however, for some therapists, their reputation, communication style, flexibility, and willingness to adapt to the client’s needs can influence the efficacy of their treatment. The CBICM is based on the principle that the strength of an intervention is the result of its influence components [38,40]. Moreover, each of these influence components exists within different parts of the communication process such as those attributed to the source, message, how the message is expressed, and whether the message can be tailored with audience feedback. Given that numerous influence techniques require audience feedback and that social media campaigns are primarily based on two-way communication, the CBICM offers a circular communication model that also describes either one-way or two-way interventions or campaigns. The CBICM was developed for this meta-analysis and is described within prior publications [34,35]. See the Multimedia Appendix for a brief overview of the CBICM.

### Study Objectives 

This meta-analysis assessed online intervention features that can be used to guide the development of population-wide campaigns targeting voluntary lifestyle behaviors. Furthermore, it assessed relationships proposed under the law of attrition, which offers insights into the role of intervention exposure (dose) and intervention efficacy. Toward these objectives, the study assesses psychological design factors, time trends, and the role of dose in online interventions.

## Methods

### Searching

To identify qualifying studies for this meta-analysis, a 3-step systematic review approach was used [51]. First, a pilot search was conducted to assess and finalize keywords and bibliographic databases. Next, query terms were constructed from keyword combinations across three categories, including spelling variations. The three keyword categories include (1) *online media terms*: internet, online, on-line, web, website, webpage, web-based, www, cyber, cyberspace, hypertext, email, e mail, and e-mail; (2) *intervention terms*: intervention and interventions; and (3) *behavioral outcome terms*: behavior, behaviour, behavioral, and behavioural. To combine these keyword categories, the first query combined online media and intervention terms; the second, online media and behavioral outcome terms. The syntax was as follows: (word category 1 AND word category 2) OR (word category 1 AND word category 2) OR (etc). These combinations produced 84 separate queries.

Second, these terms were used to identify and retrieve abstracts from relevant databases. In all, 5 bibliographic databases were selected. To cover the timeframe from 1999 through 2008, these databases were searched on September 20, 2008, and then on January 16, 2009, to cover 2008. The outcomes from both search sessions resulted in the following number of potential studies: 652 from Web of Knowledge, 292 from PsycINFO, 244 from MEDLINE, 327 from PubMed, and 7 from the Cochrane Library.

Third, additional strategies were employed to identify potential studies from the gray literature. A total of 59 additional studies were retrieved from the bibliographies of similar meta-analyses [9,10,52]. Further, requests for suitable publications were sent to relevant online discussion forums. These included listservs for the Georgetown University social marketing group, Community Based Social Marketing, Association of Internet Researchers, and the Medicine 2.0 Conference discussion group. For gray literature, searches were undertaken in Google and Yahoo. These strategies produced 6 additional papers.

### Selection 

Eligible studies for this meta-analysis included published or unpublished research and reports in English. Qualifying papers included experimental, quasi-experimental, and correlational studies, including those with randomized and nonrandomized allocations. The substantive criteria in Table 1 were used to screen studies that reflected audiences and behaviors similar to those targeted by social marketing campaigns and studies where effect sizes statistics could be extracted.

**Table 1:** Inclusion and exclusion criteria

Area	Criteria
Timeframe	Inclusion: Years 1999 through 2008
Age	Inclusion: Preteens to older persons Exclusion: Studies containing persons 9 years and younger
Behavioral domains	Inclusion: Health, safety, environmental, and community development behaviors Borderline inclusion: Subjects with ailments for which the behavior was beneficial but not critical and occupational groups for which the target behavior was voluntary Exclusion: Compulsory behaviors, critical behaviors linked to chronic illness, and psychological disorders
Behavioral outcome (dependent variable)	Inclusion: A clear behavioral change outcome Borderline inclusion: Interventions that blended change with maintenance objectives such as interventions encouraging both weight loss and maintenance Exclusion: Psychological outcomes and behavioral maintenance defined as *not changing*, that is, conceptually distinct from behavioral change
Intervention types	Inclusion: Web-based or Web and email-based Borderline inclusion: Interventions stored on a CD-ROM, USB stick, or intranet provided they contained an intervention designed for Internet deployment and technology, such as pedometers, provided both intervention and control groups received them so that any statistical difference was explained by the Web-based intervention, not the additional treatment
Intervention mechanism	Inclusion: Primarily automated interventions (human-computer) Borderline inclusion: Interventions that were primarily human-computer, but included minor computer-mediated communication; cases where both the experimental and control groups received similar human contact, so the difference lay with the online intervention; cases where human interaction was secondary, such as technical support, voluntary help lines, or minor councilor engagement Exclusion: Primarily computer-mediated communication (human-human)
Control treatments	Inclusion: Control group intervention comprising print, Web-based interventions, waitlists, placebos, and therapists Exclusion: Studies that contrasted different behavioral outcomes; studies where the difference between interventions was a non-Web based factor, such as contrasting populations or administering a mobile phone to one group; studies where the difference between the 2 interventions was unclear

**Figure 1:**
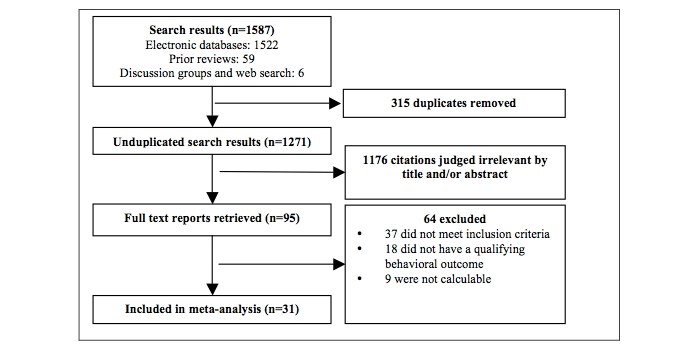
Selection process flow chart

Figure 1 shows the intervention selection process. From all sources, 1587 abstracts, references, and papers were reviewed; 315 were duplicates resulting in a pool of 1271 potentially qualifying papers. After manually reviewing titles, abstracts, and full texts, 1176 were assessed as irrelevant. For the remaining 95, the full texts were obtained and evaluated. A further 64 were excluded for not meeting the inclusion criteria, not containing a qualifying behavioral outcome, or not being suitable for calculation.

In total, 31 studies were included in this meta-analysis and coded. There were 2 studies that met the inclusion criteria that were removed from the overall analysis but were included in the dose analysis. The first study [53] was the only correlational investigation that required separate analysis [54]. The second study [55] was the only investigation that reported only a therapist control group, which could not be included in the moderator analysis as a single case.

### Validity Assessment

To evaluate the studies and test for potential publication bias, three validity assessment methods were employed: research quality assessment, cumulative meta-analysis, and a funnel plot assessment [56]. First, as the inclusion criteria covered both experimental and correlational studies, research quality was assessed with the Downs and Black instrument for randomized and nonrandomized studies [57], a checklist of 27 items pertaining to reporting, external validity, internal validity, and selection bias. However, the one item on statistical power was removed, as this factor is addressed by the meta-analysis weighting. This assessment instrument was highly rated in a review of research evaluation tools [58]. No minimum research quality score was used to screen studies, but rather, the quality score was used to assess whether research quality may have biased the pool of studies. A meta-regression analysis showed a small statistically insignificant positive correlation between research quality and effect size where k refers to the number of interventions used in the analysis (*r* = .116, *P* = .55, k = 30). This indicates that research quality is probably not correlated with effect size. However, one quasi-experimental study [59] required special treatment as it scored lowest on the research quality assessment but had the largest population.

Second, a cumulative meta-analysis did not show that small studies were contributing a large impact on the final effect size. Thus, the small studies are unlikely to be biasing the sample of studies [56].

**Figure 2:**
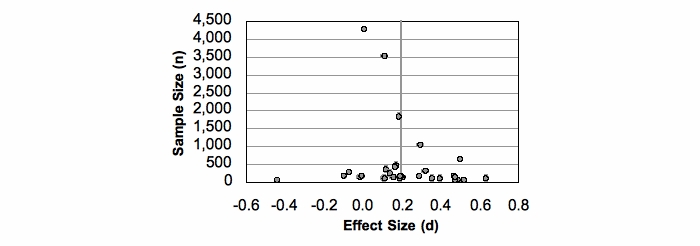
Funnel plot of interventions

Third, the funnel plot in Figure 2 displays interventions arranged with sample size on the y-axis and effect size on the x-axis. In the absence of publication bias, studies should spread out evenly around the combined effect [60]. To assess publication bias, a manual check was made; two issues were found. There is a significant discrepancy between large and small studies: 4 studies had sample sizes over 1000, while the remainder were significantly smaller. Further, the study with the largest sample size (and lowest research quality score) did not line up as would be expected in an ideal funnel plot distribution. The funnel plot suggests the sample of studies is not fully ideal, indicating some bias, but appears acceptable.

Publication bias is conventionally assessed according to three categories: *trivial*, which does not change the results; *modest*, where the results change, but the conclusions stay the same; or *substantial*, where the conclusions may be called into question [56]. This analysis revealed 2 possible sources of bias: a less than ideal distribution of interventions (indicated by the funnel plot) and the impact of one study (with the largest population size and lowest research quality score). Given the random effects model used for this meta-analysis, these possible sources of bias do not change the final statistical outcomes by more than a small margin. The small potential bias seems modest and unlikely to alter the conclusions, though in one case, the suspect study has been given special consideration.

### Data Abstraction

Data was extracted from studies using calculations by Borenstein et al and Lipsey and Wilson [56,61]. When the reported data was insufficient for coding, procedural work-arounds were used [61]. When it was impossible to code qualifying papers, a request for data was sent to the authors. For each effect size, only one outcome measure was selected per independent intervention sample [61]. When more than one follow-up measure was reported, these were also coded for the longitudinal analysis, which was analyzed within separate time groupings to avoid dependence [56]. Additionally, when more than one behavioral outcome was reported, if they were dissimilar or measured on different scales, the most relevant outcome was selected, and if several similar outcomes were reported and measured on the same scale, they were pooled. When interventions targeted multiple behaviors, a single outcome that best reflected both behaviors was selected. Coding was carried out by a single researcher who conducted the initial coding and then 1 month after completing all papers, conducted a second confirmatory coding.

For the analysis of psychological design, the CBICM was used as a framework to group influence components from various influence systems. When coding influence components, 2 approaches were used. First, *absolute coding* describes when an intervention uses an influence component whether or not the control group received the same treatment. Absolute coding is used for descriptive statistics and shows how often a particular influence component was used. Second, *relative coding* records when a particular influence component was only administered to the experimental group. If an influence component was applied to both the experimental and control groups, then the component was not coded, as it could not statistically explain the psychological difference between treatments. Relative coding is used to calculate associations between influence components and behavioral outcomes.

For the dose analysis, when coding the adherence variables, study adherence was measured as the percentage of participants in a study at a given time compared with the baseline. Coding intervention adherence was more challenging, as it was conceived and reported in many ways. Across studies, intervention adherence was reported as log-ins, visits, page views, core pages viewed, percent of required reading completed, and complex multi-item measures. Researchers reported intervention adherence by the total number of users, averages per user, or percentages over various time units. In some cases, the variables were measured on continuous scales, in others, they were dichotomous, but more often, continuous variables were cut into arbitrary categories, such as high/low log-in groups. To deal with this diversity, 2 coding and meta-analytical approaches were employed to assess the relationship between intervention adherence and behavioral outcomes. The first approach coded any reported intervention adherence construct, while the second approach only coded adherence constructs that could be converted into a percentage.

Full intention to treat groups may distort the results by including many unmotivated participants, while the fully exposed group are likely to represent the most motivated participants [32]. In aiming to keep subject groupings comparable across studies when papers reported both intention to treat and full exposure groups, the 2 were pooled to render effect size calculations more comparable with the majority of studies that did not employ these distinctions.

### Quantitative Data Synthesis

This study presents three analyses. The first analysis provides the overall effect size estimates, including groupings by control conditions and time moderators. The second analysis assesses psychological design features, presenting overall correlations, descriptive statistics, and behavioral outcomes associated with influence components. The third analysis examines correlations between adherence variables and behavioral outcomes.

Following recommendations to select statistical models a priori on the basis of substantive justifications [51,56], a random effects model was selected. Intervention effect size, standard error, and inverse variance statistics were calculated with equations and the spreadsheet tool by Lipsey and Wilson [61]. Overall effect sizes and analogue to analysis of variance (ANOVA) analyses were carried out in comprehensive meta-analysis. Meta-regression was conducted in SPSS, version 14 (SPSS Inc, Chicago, IL) with macros using maximum likelihood [61].

The majority of studies were randomized controlled trials, measured with continuous or dichotomous data with pre and post measures, while in some cases only post measures were reported. For group contrasts, that is, between-subject studies, the standardized mean difference, d, was used as the primary effect size measure. To assess categories used to explain heterogeneity in the analogue to ANOVA, the between-group heterogeneity statistic and its significance value Q_b_ (*P*) are used to assess the strength of the categories. Likewise, the within-group heterogeneity statistics Q_w_ (*P*) and I^2^ are used to assess the strength of categories [51,56]. As standard notation, *r* designates meta-regression correlations, and k, the number of interventions.

## Results

### Study Characteristics

Table 2 lists the 30 interventions from 29 studies that qualified for the primary analysis. One study contained 2 interventions, which are designated as *a* and *b* [62]. Across these studies, 17,524 participants were allocated to 30 interventions, with 14,895 participants completing postintervention surveys. Of the interventions, 24 used random assignment, 1 was nonrandom, and it was not possible to determine the type of assignment for 5 interventions.

Table 2 presents the pre and post number of subjects across the experimental and control groups. For the experimental group, Table 2 presents the mean age, the percentage of male participants, study adherence, and intervention adherence (recorded at first postintervention measure). Finally, the research score is presented as a percentage.

**Table 2:** Interventions

Author (Year) and Reference Number	Experimental and Control Groups		Experimental Group					Research Score (%)
	Pre (n)	Post (n)	Participant Characteristics	Mean Age	Male (%)	Study Adherence (%)	Intervention Adherence (%)	
Bersamin et al (2007) [63]	139	139	Students (who drink alcohol)	18	48.0%	57.4%		73.1%
Bewick et al (2008) [64]	506	317	Students	21.3	31.0%	59%		73.1%
Bruning Brown et al (2004) a [62]	153	153	Students (female)	15.1	0.0%	66.7%		69.2%
Bruning Brown et al (2004) b [62]	69	69	Parents		3.4%	100.0%	50.0%	69.2%
Celio et al (2000) [65]	52	47	Students (female)	19.6	0.0%	96.3%	71.0%	92.3%
Chiauzzi et al (2005) [1]	265	215	Students (who are heavy drinkers)	20	44.8%	80.2%	86.0%	80.8%
Dunton and Robertson (2008) [66]	155	128	Women	42.8	0.0%	78.6%		92.3%
Gueguen and Jacob (2001) [67]	1008	1008	French citizens					61.5%
Hunter et al (2008) [68]	451	446	Military personnel	33.5	50.0%	85.0%		80.8%
Jacobi et al (2007) [69]	97	97	Students (female)	22.5	0.0%	100.0%	83.0%	80.8%
Kim and Kang (2006) [70]	50	50	Diabetics	55.1	53.4%			73.1%
Kosma et al (2005) [71]	151	75	Disabled persons			45.5%		84.6%
Kypri et al (2004) [72]	104	83	Students	19.9		82.4%	100.0%	76.9%
Kypri and McAnally (2005) [73]	146	122	Students	20.3	46.0%	82.0%	100.0%	76.9%
Lenert et al (2004) [74]	485	144	Smokers	39	42.0%	26.0%		57.7%
Marshall et al (2003) [75]	655	258	University faculty and staff	43	50.0%	76.5%	26.0%	73.1%
McConnon et al (2007) [3]	221	131	Obese persons	45.8	23.0%	48.7%	53.0%	76.9%
McKay et al (2001) [76]	78	68	Diabetics	52.3	18.0%	92.1%		84.6%
Moore et al (2005) [77]	100	100	Students	21.7	42.2%	86.2%		65.4%
Napolitano et al (2003) [78]	65	52	Hospital staff	42.8	16.1%	70.0%		80.8%
Oenema et al (2005) [79]	521	384	Employees	42	57.0%	72.0%		69.2%
Petersen et al (2008) [59]	4254	4254	Employees			21.2%		38.5%
Roberto (2007) [80]	378	103	Students (high school)	15.5	41.7%	84.8%	88.5%	53.8%
Severson et al (2008) [81]	2523	1801	Smokeless tobacco users	36.7	97.9%	44.1%	50.0%	57.7%
Strecher et al (2005) [2]	3501	3501	Smokers trying to quit with the nicotine patch	36.9	43.5%	46.6%		80.8%
Strom et al (2000) [82]	102	45	Headache sufferers	41.5	25.0%	39.2%		80.8%
Swartz et al (2006) [83]	351	274	Employees	40.9	46.8%	50.9%	70.2%	80.8%
Tate et al (2001) [84]	91	81	Overweight persons	40.6	11.0%	78.3%		96.2%
Verheijden et al (2004) [85]	146	130	Persons at risk of cardiovascular disease	62	72.0%	84.9%	32.9%	84.6%
Winett et al (2007) [86]	707	620	Church congregation	53.13	33.0%	88.5%	57.0%	57.7%

Table 3 shows experimental group demographics that have been weighted by pretest experimental group totals. With demographic records for 8813 pretest participants, the average age was 34.7 and weighted average age was 36.5 (k = 26, n = 6057). The age standard deviation was 6.6; the weighted average standard deviation was 9.0 (k = 21, n = 5691). In general, the balance between genders was similar, with just slightly more men. The majority were white and possessed a university degree.

**Table 3:** Demographic descriptives

Demographic Descriptives		k	n	Percent
**Gender**		26	6028	100%
	Men		3152	52.3%
	Women		2876	47.7%
**Education**		15	2341	100%
	Bachelor’s level		1347	57.6%
	Master’s level		552	23.6%
	Secondary		404	17.2%
	Primary		38	1.6%
**Descent**		19	2957	100%
	White		2475	83.7%
	African		144	4.9%
	Mixed		116	3.9%
	Asian		82	2.8%
	Latin American		74	2.5%
	Aboriginal		33	1.1%
	Unclassified		33	1.1%

### Overall Effect Size Estimates

**Figure 3:**
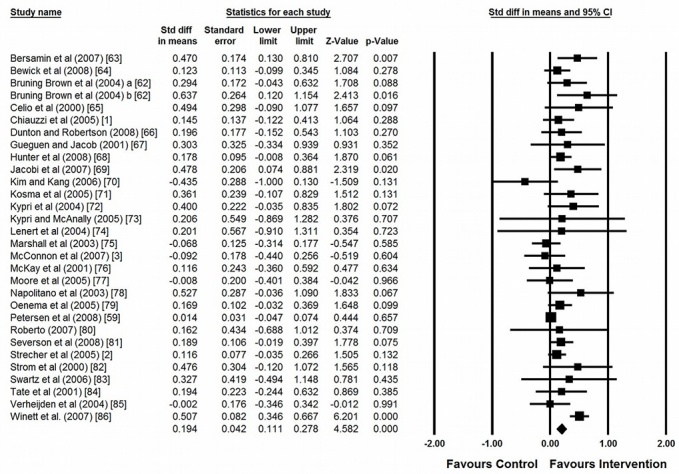
Forrest plot

Table 4 reports the primary effect sizes estimates, while the forest plot with all interventions is available in Figure 3. Query 1 used the first posttest effect size from all 30 interventions. Query 2 included all posttest effect sizes, resulting in 38 effect sizes across 3 timeframes.

**Table 4:** Effect size estimates

Groupings		k	d (95% confidence interval [CI])	*P*	Q_b_ (*P*)	Q_w_ (*P*)	I^2^
**Overall effect size**^a^		30			N/A		
	All interventions	30	0.194 (0.111 - 0.278)	< .001		64.125 (< .001)	54.776
**Control group**^a^		30			9.109 (.01)		
	Waitlist or placebo	18	0.282 (0.170 - 0.393)	< .001		55.163 (< .001)	69.183
	Website	8	0.162 (0.006 - 0.318)	.04		0.650 (.10)	< 0.001
	Print	4	-0.110 (-0.343 to 0.123)	.35		1.623 (.65)	< 0.001
**Intervention duration**^a^		30			6.611 (.16)		
	Single-session	4	0.404 (0.130 - 0.677)	.004		0.367 (.95)	< 0.001
	From 2 days to 1 month	5	0.205 (0.026 - 0.383)	.024		4.511 (.34)	11.336
	Over 1 month to 4 months	16	0.220 (0.116 - 0.324)	< .001		30.131 (.01)	50.218
	Over 4 months to 7 months	3	0.090 (-0.077 to 0.258)	.29		3.235 (.20)	38.186
	Over 7 months to 13 months	2	-0.047 (-0.337 to 0.243)	.75		0.130 (.72)	< 0.001
**Long-term impacts**^b^		38			N/A		
	From 1 day to 1 month	24	0.194 (0.107 - 0.282)	< .001		39.329 (.02)	41.519
	Over 1 month to 4 months	10	0.226 (0.089 - 0.363)	.001		7.139 (.62)	< 0.001
	Over 4 months to 7 months	4	0.157 (0.002 - 0.312)	.048		15.261 (.002)	80.342

^a^ Query 1

^b^ Query 2

Table 4 shows the overall effect size, which is small and statistically significant. However, the various interventions are not likely to represent a single homogenous group, as indicated by the 2 within-group heterogeneity statistics Q_w_ (*P*) and I^2^ that show a level of heterogeneity that cannot be explained by sampling error alone. Control group comparisons provide the best way to model the heterogeneity across interventions, as indicated by the significant between-group heterogeneity statistic Q_b_ (*P*) that was less than .05, revealing a large difference between control group categories. In general, online interventions showed the largest effect size when compared with waitlists and placebos, a smaller effect when compared with lower-tech online interventions, and a negative statistically insignificant effect size when compared with print interventions.

**Figure 4:**
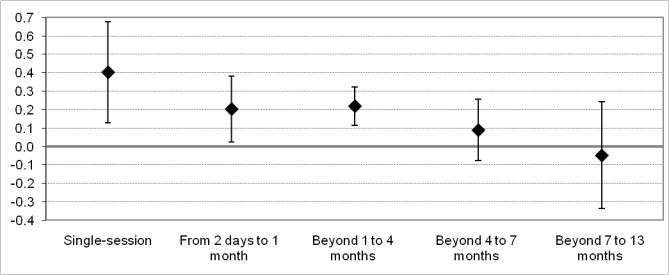
Effect Size by intervention duration

The figures for intervention duration are presented in Table 4 and Figure 4. The results suggest that shorter interventions offer larger impacts, while longer interventions offer lower impacts. The strongest effect sizes resulted from the single-session interventions. Interventions lasting up to 4 months provided an effect size close to the overall effect size. However, interventions that operated longer than 4 months were statistically insignificant, demonstrating no substantial behavioral impact.

To examine the long-term impact after an intervention had ended, all postintervention measures were grouped into 3 time categories. This resulted in the 38 distinct postintervention measures; these are referred to as Query 2 in Table 4. As it is only possible to analyse 1 measure from each intervention sample, no between-group heterogeneity analysis was undertaken. In general, the long-term impact appears to last several months. The pooled effect size of the 24 interventions in the first time frame is similar to the overall effect size. The effect size rises slightly from 1 to 4 months and then drops slightly for the final postintervention measure, from 4 to 7 months.

### Psychological Design 

This section presents two analyses of psychological design. The first assesses the relationship between the overall psychological design and behavioral outcomes. The second analysis presents the psychological architecture of online interventions, reporting how frequently influence components are used and their associated effect sizes.

Of the theories used to design interventions, the transtheoretical approach was the most popular, being used across 47% (14/30) of the interventions. Other theories used to design interventions included social cognitive (4/30, 13%), cognitive behavioral therapy (4/30, 13%), behavioral therapy (3/30, 10%), extended parallel process model (2/30, 7%), health belief model (2/30, 7%), and the theory of reasoned action (2/30, 7%).

#### Psychological Design: Overall Correlations

This section assesses relationships between an intervention’s overall psychological architecture and its effect size. The analysis is based on the coding systems of behavioral change techniques [38] and of behavioral determinants [50], which were relative coded in order to assess influence components administered to the experimental group only.

**Figure 5:**
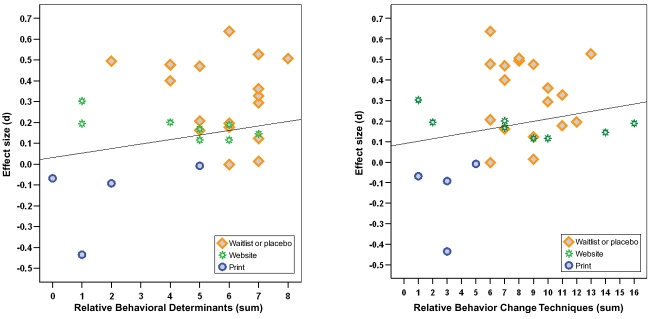
Sum of influence components by effect size

Groups of online interventions with the largest number of influence components demonstrated the largest effect sizes. Nonetheless, statistical correlations between influence components and effect size were inconclusive. Figure 5 compares effect sizes with the sum of relative influence components for two clusters: the first, behavioral determinants, and the second, behavior change techniques. The trend line is derived from the meta-regression analysis. Each intervention is clustered according to its control condition. Interventions matched against waitlist or placebo control groups achieved the highest effect sizes and contained the largest number of relative influence components (average of 5.7 behavioral determinants and 8.6 behavior change techniques). Interventions compared with website control groups attained a smaller but significant effect size and possessed fewer influence components (average of 4.4 behavioral determinants and 8.3 behavior change techniques). Finally, interventions compared with the sophisticated print intervention control groups were statistically no different from print publications and possessed the fewest influence components (average of 2 behavioral determinants and 3 behavior change techniques).

Meta-regression demonstrated a moderate but statistically insignificant correlation between an intervention’s total influence components and their effect size. However, there are reasons to suspect an association exists nonetheless. The meta-regression correlation between the sum of behavior change techniques and effect size is (*r* = .219, *P* = .26, k = 30), and the correlation between the sum of behavioral determinants and effect size is (*r* = .327, *P* = .09, k = 30). Although these meta-regression analyses demonstrated no statistically significant correlation, the following evidence suggests a relationship. The groups of interventions with the largest number of influence components achieved the largest outcomes, while the groups of interventions with fewer influence components achieved lower behavioral impacts. Moreover, the same calculations were conducted without the study [59] that was shown to be suspect in the validity assessment. After removal, the correlation between behavior change techniques and effect size remained statistically insignificant. However, the relationship between behavioral determinants and effect size was large and statistically significant (*r* = .470, *P* = .007, k = 29).

#### Psychological Design: Influence Component Frequency and Effect Sizes

This section uses the CBICM as a framework to describe the psychological architectures employed by online interventions. Influence components are clustered within the social context, media channel, feedback message, source interpreter, source encoding, intervention message (behavior change techniques), and audience interpreter (behavioral determinants and demographics). To encourage personal change, many of the interventions helped participants adopt healthy habits by motivating them to set goals, record their behavior, learn new skills, and then use feedback to track their progress.

*Absolute coding* describes how frequently a particular influence component is used across the 30 interventions. *Relative coding* is used to assess the pooled effect size associated with each influence component. In general, the absolute coding number of interventions k is larger than the relative coding number of interventions k. This is because an intervention may have used a particular influence component, such as tailoring. However, if the control condition also used tailoring, then tailoring could not explain the statistical difference between the 2 conditions. Consequently, absolute coding provides insight into how often an influence component is used, while relative coding draws on a smaller number of studies to assess the effect size of each influence component. Effect sizes were only calculated where there were at least 2 qualifying studies.

The *social context* describes the social and environmental contexts that can influence an intervention’s effectiveness. The majority of interventions (15) operated through direct interaction between participants and the intervention system. A slightly smaller number (13) of interventions occurred in contexts where there was at least 1 point of contact within an institutional setting. Just 2 interventions drew on family and friends.

Table 5 presents the CBICM media channel, audience feedback message, source interpreter, and source encoding. The *media channel* describes the communication channels used to distribute an intervention. Interventions primarily combined websites with email, while a third were just Web-based. Although the results show that Web-based interventions are more effective than combined websites with email, this is likely due to the strong effect of the single-session interventions that did not use email.

The *feedback message* describes information that users send to the intervention system, which is used to design personally relevant intervention messages. Systems that do not factor user feedback into their interventions are not able to deploy these influence components. Tailoring is the most common feedback component and offered a reasonable effect size. Tailoring was frequently combined with personalization: the 12 interventions that used personalization also used tailoring. The most effective influence component was *providing feedback on performance*, which fits with the goal directed nature of these interventions, as discussed subsequently.

The *source interpreter* describes influence components that are based on audiences’ perceptions of the source, either the organizations operating the intervention or the website itself. Few studies explicitly mentioned source factors, making it difficult to reliably code the components and calculate their associated effect sizes. Nonetheless, the few interventions that demonstrated similarity to the audience members showed a strong effect size. Visually attractive design did not show any advantage, and just one study mentioned credibility factors.

*S* *ource encoding* describes how an intervention is expressed. The vast majority of interventions were source encoded as processes that engaged users through multiple interactions over time. Those interventions that occurred in a single interaction were highly effective, which is consistent with the prior trend showing that single-session interventions were most effective. Only one intervention used a sequential request technique, the *foot in the door* technique.

**Table 5:** Media channel, feedback message, source interpreter, and source encoding

		Absolute Coding		Relative Coding					
CBICM clusters		k	%	k	d (95% CI)	*P*	Q_w_ (*P*)	I^2^
**Media channel**								
	Website and email	20	66.7%	14	0.165 (0.054 - 0.276)	.004	24.914 (.02)	47.820
	Website	10	33.3%	8	0.309 (0.150 - 0.467)	<.001	16.636 (.02)	57.922
**Audience feedback message**								
	Tailoring	25	83.3%	22	0.201 (0.107 - 0.296)	<.001	53.428 (<.001)	60.695
	Provide feedback on performance	20	67.0%	18	0.215 (0.109 - 0.321)	<.001	52.985 (<.001)	67.915
	Personalization	12	40.0%	11	0.193 (0.048 - 0.337)	.009	7.651 (.66)	< .001
	Adaptation/content matching	2	6.7%	2	0.191 (-0.138 - 0.521)	.26	0.135 (.71)	< .001
**Source interpreter**								
	Attractiveness	5	16.7%	3	0.080 (-0.215 - 0.375)	.60	2.631 (.27)	23.975
	Similarity	3	10.0%	3	0.324 (0.015 - 0.632)	.04	1.078 (.58)	< .001
	Credibility	1	3.3%	1				
**Source encoding**								
	Multiple interactions	23	77.0%	16	0.208 (0.098 - 0.319)	<.001	43.657 (<.001)	65.641
	Single interaction	3	10.0%	2	0.473 (0.154 - 0.792)	.004	0.001 (.98)	< .001
	Sequential requests (foot in the door)	1	3.0%	1				

Table 6 presents influence components within the *source intervention message*, which represents the overt treatment designed to impact audience psychology and/or behavior. The taxonomy of behavior change techniques [38] is used for this cluster with *providing feedback on performance* moved to the feedback message cluster (as it can only exist when feedback mechanisms are employed). Most of the intervention messages informed users about the consequences of their behavior, focused on goal setting, and provided instructions on performing the behavior. The majority of self-monitoring was directed toward the behavior, with a few interventions focused on monitoring behavioral outcomes. However, both approaches produced similar effect sizes. Although *action planning* is a popular and effective approach, *setting graded tasks* showed no significant contribution. Both *agreeing to a behavioral contract* and *time management* stood out as influence components that were infrequently used but were associated with an above average effect size.

**Table 6:** Source intervention message (behavioral change techniques)

	Absolute Coding		Relative Coding				
Behavioral Change Techniques	k	%	k	d (95% CI)	*P*	Q_w_ (*P*)	I^2^
Provide information on consequences of behavior in general	23	76.7%	16	0.306 (0.173 - 0.438)	< .001	11.365 (.72)	< 0.001
Goal setting (behavior)	21	70.0%	16	0.245 (0.131 - 0.359)	< .001	49.984 (< .001)	69.991
Prompt self-monitoring of behavior	19	63.3%	16	0.223 (0.108 - 0.339)	< .001	52.600 (< .001)	71.483
Provide instruction on how to perform the behavior	18	60.0%	15	0.212 (0.102 - 0.323)	< .001	27.927 (.02)	49.870
Action planning	17	56.7%	13	0.240 (0.119 - 0.360)	< .001	46.702 (< .001)	74.305
Provide normative information about others’ behavior	12	40.0%	12	0.246 (0.120 - 0.373)	< .001	6.893 (.81)	< 0.001
Fear arousal	12	40.0%	10	0.193 (0.042 - 0.344)	.01	6.491 (.69)	< 0.001
Barrier identification/problem solving	10	33.3%	10	0.224 (0.076 - 0.372)	.003	4.372 (.89)	< 0.001
Provide information on where and when to perform the behavior	10	33.3%	10	0.218 (0.095 - 0.340)	< .001	20.104 (.02)	55.232
Set graded tasks	10	33.3%	9	0.095 (–0.017 to 0.207)	.10	11.464 (.18)	30.216
Plan social support/social change	9	30.0%	5	0.250 (0.035 - 0.465)	.02	1.940 (.75)	< 0.001
Facilitate social comparison	9	30.0%	9	0.226 (0.070 - 0.382)	.004	4.439 (.82)	< 0.001
Model/demonstrate the behavior	8	26.7%	8	0.210 (0.056 - 0.365)	.008	3.886 (.79)	< 0.001
Provide information on consequences of behavior relevant to the individual	8	26.7%	8	0.208 (0.040 - 0.375)	.02	3.447 (.84)	< 0.001
Environmental restructuring	7	23.3%	4	0.189 (–0.028 to 0.406)	.09	1.229 (.75)	< 0.001
Prompt review of behavioral goals	7	23.3%	7	0.138 (–0.018 to 0.294)	.08	6.888 (.33)	12.887
Agree behavioral contract	5	16.7%	4	0.275 (0.105 - 0.446)	.002	13.001 (.005)	76.925
Prompt self-monitoring of behavioral outcome	5	16.7%	5	0.263 (0.080 - 0.446)	.005	36.961 (< .001)	89.178
Prompt identification as role model/position advocate	5	16.7%	4	0.078 (–0.107 to 0.263)	.41	1.738 (.63)	< 0.001
Time management	4	13.3%	4	0.343 (0.018 - 0.669)	.04	1.476 (.69)	< 0.001
Stress management	4	13.3%	4	0.185 (–0.009 to 0.380)	.06	1.517 (.68)	< 0.001
Prompt self talk	3	10.0%	3	0.319 (0.058 - 0.581)	.02	2.168 (.34)	7.747
Provide rewards contingent on successful behavior	3	10.0%	3	0.291 (0.023 - 0.560)	.03	1.478 (.48)	< 0.001
Provide information about others’ approval	3	10.0%	3	0.206 (–0.040 to 0.453)	.10	0.461 (.79)	< 0.001
Use of follow-up prompts	3	10.0%	3	0.183 (–0.098 to 0.463)	.20	0.968 (.62)	< 0.001
Goal setting (outcome)	3	10.0%	1				
Relapse prevention/coping planning	3	10.0%	2	0.149 (–0.100 to 0.398)	.24	0.310 (.58)	< 0.001
Shaping	3	10.0%	3	0.091 (–0.236 to 0.418)	.59	0.524 (.77)	< 0.001
General communication skills training	2	6.7%	2	0.295 (–0.031 to 0.622)	.08	2.737 (.10)	63.458

Emotional control training	2	6.7%	2	0.253 (–0.061 to 0.568)	.11	.796 (.37)	< 0.001
Prompting focus on past success	1	3.3%	1				
Prompt use of imagery	1	3.3%	1				
Motivational interviewing	1	3.3%	1				
Prompting generalization of a target behavior	1	3.3%	1				
Provide rewards contingent on effort or progress toward behavior	1	3.3%					
Teach to use prompts/cues	1	3.3%					
Prompt anticipated regret	0	0%					
Prompt practice	0	0%					
Prompt review of outcome goals	0	0%					

The *audience interpreter* describes the demographic disposition and psychology of the individual or population targeted to adopt a behavior. In the CBICM, this is where audience demographics are clustered. This is also where behavioral determinants are grouped—these are the psychological constructs believed to directly influence behavior.

Table 7 presents the demographic moderators for participants’ age and gender*.* Both groups were divided into 3 equal categories, and then effect sizes were calculated for each group. Across both age and gender groupings, the overall between-group heterogeneity statistics Q_b_ (*P*) was greater than .05, indicating that the categories were quite similar and did not explain the heterogeneity. Among the 3 age groups, interventions with younger audiences (average age 15 to 21.4 years) tended to achieve the largest outcomes, followed by middle-aged (average age 21.5 to 41.8 years), and finally older participants whose average age was greater than 41.9 achieved the lowest outcomes with statistically insignificant results. For the gender groupings, the intervention group with more females showed greater impact than the mixed gender group, and a far larger impact than the statistically insignificant male-dominated group.

**Table 7:** Demographic moderators

Groupings		k	d (95% CI)	*P*	Q_b_ (*P*)	Q_w_ (*P*)	I^2^
**Age Groups (years)**		30			1.248 (.74)		
	Younger (15.0 - 21.4)	8	0.271 (0.095 - 0.446)	.002		4.676 (.70)	< 0.001
	Middle (21.5 - 41.8)	9	0.198 (0.045 - 0.352)	.01		4.725 (.79)	< 0.001
	Older (41.9 and over)	9	0.141 (–0.003 to 0.286)	.06		29.017 (< .001)	72.430
	Unknown	4	0.190 (–0.033 to 0.414)	.10		8.196 (.04)	63.397
**Gender groups**		30			5.889 (.12)		
	More female (66.6% - 100%)	12	0.307 (0.187 - 0.427)	< .001		18.290 (.08)	39.857
	Mixed	12	0.122 (0.010 - 0.235)	.03		11.354 (.41)	3.116
	More male (66.6% - 100%)	2	0.123 (–0.111 to 0.357)	.30		0.864 (.35)	< 0.001
	Unknown	4	0.124 (–0.049 to 0.297)	.16		5.685 (.13)	47.233

Table 8 shows the audience’s behavioral determinants targeted by interventions. These are the psychological constructs employed by various behavioral change theories. The coding is based on the list of behavioral determinants [50]. The psychological architecture of the websites resembled behaviorist-type therapies where the focus was on knowledge, awareness of risks, goal setting, and skill building. Across interventions, knowledge was the most common and effective behavioral determinant, while emotional appeals alone were used by a third of interventions and were associated with a lower effect size. Similarly, skill building offered an effective influence component, while self-efficacy was surprisingly low. One noteworthy exception is the strong contribution of social norms, which was both common and effective. The least frequent behavioral determinant was an appeal to the participant’s social-professional role or identity, which on its own, was an exclusion criteria in this study.

**Table 8:** Audience interpreter (behavioral determinants)

	Absolute Coding		Relative Coding				
Behavioral Determinants	k	%	k	d (95% CI)	*P*	Q_w_ (*P*)	I^2^
Knowledge	30	100.0%	16	0.291 (0.166 - 0.416)	< .001	53.257 (< .001)	71.835
Motivation and goals (intention)	26	86.7%	20	0.229 (0.129 - 0.329)	< .001	54.332 (< .001)	65.030
Social influences (norms)	22	73.3%	18	0.250 (0.147 - 0.354)	< .001	52.042 (< .001)	67.334
Beliefs about consequences	21	70.0%	19	0.268 (0.182 - 0.353)	< .001	23.034 (.19)	21.855
Skills	19	63.3%	15	0.185 (0.069 - 0.300)	.002	46.753 (< .001)	70.055
Memory, attention, and decision processes	18	60.0%	17	0.188 (0.080 - 0.297)	.001	42.480 (< .001)	62.335
Behavioral regulation	17	56.7%	14	0.218 (0.103 - 0.332)	< .001	40.971 (< .001)	68.270
Emotion	10	33.3%	9	0.183 (0.026 - 0.341)	.02	6.966 (.54)	< 0.001
Nature of the behaviors	9	30.0%	6	0.274 (0.137 - 0.411)	< .001	16.142 (.006)	69.024
Beliefs about capabilities (self-efficacy)	8	26.7%	7	0.083 (–0.051 to 0.218)	.23	4.545 (.60)	< 0.001
Environmental context and resources	6	20.0%	3	0.180 (–0.044 to 0.404)	.12	1.060 (.59)	< 0.001
Social-professional role and identity	3	10.0%	2	0.275 (–0.321 to .871)	.37	0.024 (.88)	< 0.001

### Dose (Adherence and Attrition)

**Figure 6:**
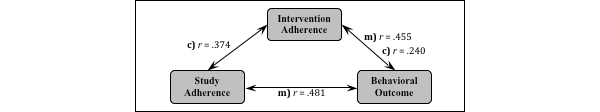
Correlations between adherence variables and effect size (c = correlation effect size, m=meta-regression effect size)

To assess correlations among the 3 dose variables (intervention adherence, study adherence, and behavioral outcomes), 2 meta-analytical methods were employed and combined in Figure 6. The analyses show a significant correlation between study adherence and intervention adherence and a significant correlation between study adherence and behavioral outcomes. However, the two methods produced one contradictory result, with one method showing the association between intervention adherence and outcome to be statistically significant, and the other, insignificant. Though, for methodological reasons, the association is likely to be significant.

The first analysis pooled correlation effect sizes; is designated *c* in Figure 6. This analysis included 2 papers that qualified for the dose analyses [53,55] but which were excluded from the primary investigation. Only 5 studies were used to assess the relationship between study adherence and intervention adherence. However, the association was strong and significant (r = .374, 95% CI = .246 to .489, *P* < .001, k = 5). Similarly, the relationship between intervention adherence and behavioral outcomes was modest, yet significant (*r* = .240, 95% CI = .133 - .341, *P* < .001, k = 9).

In Figure 6, the second meta-regression method uses *m* to designate the two meta-regression effect size calculations. The heavily dichotomized data used for this analysis is based on the adherence percentages presented in Table 2. This analysis shows a moderate and significant relationship between study adherence and behavioral outcomes (*r* = .481, *P* = .006, k = 28). It also showed a moderate but statistically insignificant association between intervention adherence and behavioral outcomes (*r* = .455, *P* = .109, k = 13).

Despite the two contradictory conclusions, there are compelling reasons why the relationship between intervention adherence and effect size is probably significant. Although the insignificant meta-regression analysis drew from more studies, the analysis was based on data that was heavily dichotomized, which is known to underestimate effect sizes [61]. Conversely, the significant correlation effect size drew from fewer studies with the advantage of including statistics that are closer to the original raw figures. Given the strong but insignificant correlation from the meta-regression (known to underestimate effect sizes) and the moderate and statistically significant correlation effect size analysis, it is likely that both intervention adherence and behavioral outcomes are related.

Table 9 presents the adherence averages presented in Table 2, which were used in the meta-regression dose analysis. These figures offer an explanation for the relationships between dose variables. The adherence percentage is given with a simple average and weighted average based on the posttest experimental group sample size. As the duration of an intervention increases, behavioral outcomes decrease, intervention adherence decreases, and study adherence roughly follows a downward trend with some variations.

**Table 9:** Intervention duration, adherence, and behavioral outcomes

	Study Adherence			Intervention Adherence			Behavioral Outcomes	
Intervention Duration	k	Average %	Weighted Average %	k	Average %	Weighted Average %	k	d (95% CI)
Single-session	3	73.9%	72.9%	2	100.0%	100.0%	4	0.404 (0.130 - 0.677)
From 2 days to 1 month	5	76.8%	74.4%	2	68.0%	79.8%	5	0.205 (0.026 - 0.383)
Over 1 month to 4 months	15	67.9%	53.6%	7	63.7%	53.4%	16	0.220 (0.116 - 0.324)
Over 4 months to 7 months	3	61.5%	28.1%				3	0.090 (–0.077 to 0.258)
Over 7 months to 13 months	2	66.8%	68.0%	2	43.0%	42.3%	2	–0.047 (–0.337 to 0.243)

## Discussion

The overall impact of online interventions is small, with the control conditions explaining much of the variance across studies. This suggests that online intervention efficacy should be regarded as a relative advantage in comparison to different intervention media. The largest impact was exerted from online interventions when compared with waitlists and placebos, followed by comparison with lower-tech online interventions; no significant difference was found when compared with sophisticated print interventions. In other words, online interventions offer a small effect and are probably as good as print interventions but with the advantage of lower costs and larger reach.

As a general guideline, an effect size d can be considered small (d ≤ 0.2), medium (d = 0.5), or large (d ≥ 0.8). Likewise, correlation effect sizes *r* can be considered small (*r* ≤ 0.1), medium (*r* = 0.25), and large (*r* ≥ 0.4) according to Cohen as cited by Lipsey and Wilson [61]. By Cohen's criteria, the overall results of this meta-analysis are small (d = 0.194, 95% CI = .111 - .278, *P* < .001, k = 30). However, this figure is consistent with other meta-analyses of online interventions. One comparison of 5 Web- and non-Web-based interventions produced effect sizes on knowledge and behavior (d = –0.24 to 0.44, k = 5) [10]. Another study showed effect sizes, from the first measurement, on physical activity (d = 0.05, 95% CI = –0.05 to 0.15, k = 11); weight loss (d = 0.10, 95% CI = –0.11 to 0.29, k = 8); and tobacco use (d = 0.33, 95% CI = 0.08 - 0.59, k=11) [9]. Still another showed an overall Hedges’ g effect size (d = 0.16, 95% CI = 0.09 - 0.23, *P* < .001) [8].

Time proved to be a critical factor with shorter interventions achieving the largest impacts. In general, as the length of an intervention increased, behavioral impacts and intervention adherence decreased. When examining the long-term impacts after interventions had ended, the impact appeared to increase from 1 to 4 months and then decline afterwards. These trends may be partially explained by the relationship between adherence and behavioral outcomes, where the shortest interventions achieved both the highest behavioral impacts and also the highest levels of adherence. Discussed below, this trend is proposed to be a function of decreasing motivation.

### Psychological Design

Many of the interventions appeared to be simple but, in fact, were highly complex programs that used tailoring algorithms and which in some cases, contained libraries with potentially hundreds of messages that could offer thousands of message combinations. When designing interventions, the transtheoretical approach was the most popular theory used. Interventions were primarily goal orientated. In general, the interventions in this study informed users about the consequences of their behavior, encouraged them to set goals, then encouraged them to track their progress toward those goals while providing feedback on their performance. Popular behavioral determinants targeted by these interventions included knowledge, motivation, and social norms. Regarding demographics, younger audiences achieved the largest behavioral impacts, with impact strength decreasing as participants increased in age. Female dominated groups achieved larger behavioral outcomes in comparison with mixed gender and male dominated groups. Most interventions used feedback mechanisms, with 83% using tailoring, while the 40% that used personalization also combined it with tailoring. The most effective feedback mechanism was providing feedback on performance. Source factors were rarely reported; however, interventions that reflected similarity with users demonstrated efficacy. Just one intervention reported source credibility even though credibility has been recommended by numerous design guidelines [36,87,88].

Influence components approaches [38,40,89] posit that the strength of an intervention is a function of its psychological components. This meta-analysis did not find conclusive support for this assertion, but the evidence suggests a likely trend. The inconclusive findings may be due to three factors: coding limitations, the moderate number of qualifying studies, and a potentially nonlinear relationship.

First, accurate relative coding of influence components could only take place when authors described the experimental and control groups in equal detail. Many authors did not fully describe control conditions, resulting in an overestimate of relative influence components, which may have caused measurement distortions. Additionally, interventions using stages of change frameworks tended to report a large number of influence components. However, depending on participants’ stage, they would likely be exposed to a smaller number of influence components, resulting in an overestimate in the number of relative influence components. 

Second, the strong and statistically insignificant correlations found in this study suggest that this relationship may require a larger pool of studies to overcome measurement distortions. For instance, Webb et al [8] drew on a larger pool of studies and found a statistically significant correlation. 

Third, the relationship may not be linear but rather resemble an inverted u-shaped parabola curve. For example, one research team argued that websites that provide fewer individually tailored features may be more effective in promoting and maintaining behavior than ones that offer numerous poorly presented strategies [25]. If the relationship is nonlinear, few influence components may be too few to significantly influence behavior. Too many may potentially overwhelm users with complex and demanding interventions, while there is probably a middle ground where a small number of relevant (and mutually reinforcing) influence components are most effective.

Through absolute and relative coding, it was possible to examine an influence component’s frequency of use and associated effect sizes. In general, the frequency of use demonstrated a loose association with effect size. For instance, the most commonly used influence components were often the most effective ones, though there were exceptions to this rule. This suggest that, in general, intervention researchers are probably drawing from common approaches that have been proven to work, with a smaller amount of experimental work assessing less conventional approaches.

### Dose

The law of attrition posits that study adherence and intervention adherence are likely to be correlated because they are impacted by a third variable, participant interest [32]. This assertion is somewhat supported by the results of the meta-analysis. Despite one contradictory relationship, the results suggest the relationship is likely to include 3 variables: study adherence, intervention adherence, and behavioral outcomes.

Instead of hypothesizing that attrition is a function of loss of participant interest, a slightly different proposal is that adherence is a function of participant’s motivation. By explaining the correlations as the result of motivation, this explains participant’s interest (in the terms of goal commitment) but also a second construct that encompasses ability and/or efficacy. Across different research, motivation generally encompasses these two dimensions: goal commitment and either self-efficacy or ability [90-93].

The law of attrition further proposes that study and intervention adherence follow a systematic pattern declining over time, similar to an inverse s-shaped diffusion curve [32], which can be found in the logarithmic shaped relapse curves of smokers [94]. In this meta-analysis, effect sizes, study adherence, and intervention adherence generally depreciated over time, indicating a downward trend consistent with the law of attrition.

### Practitioner and Research Implications

Intervention length proved to be a critical factor, with shorter interventions generally achieving the largest impact and intervention impact fading as an intervention's length increased. This has implications for intervention designers who need to make interventions as short as possible to cope with rapid attrition and the probable loss of motivation over time. Moreover, for some behaviors, highly tailored single-session interventions produced the strongest effect sizes. This suggests that short and tailored interventions can be as effective, if not more effective, than some longer and demanding ones. However, this trend is likely to be limited to particular behaviors, such as responsible drinking [63,72] and diet choices [63], but is less applicable to demanding change processes, such as tobacco cessation or weight loss.

Adherence variables demonstrated correlations with behavioral outcomes. This has implications for practitioners who generally seek to maximize behavioral impacts and researchers who must subject study participants to adequate dosage levels in order to conduct sound studies. To increase an intervention’s efficacy, it may be possible design adherence systems that encourage higher levels of intervention adherence. In some cases, interventions did not explicitly implement measures to maximize participant adherence, with 1 intervention attaining a median of 1 visit in 8 months [85]. At the other extreme, 1 intervention (that did not meet the inclusion criteria) encouraged users to log in at least once per week. When users did not log into the system during a given week, the systems would email them a reminder message, and if they still did not log in, the reminder was repeated the following week. After not logging in for 2 weeks, the system made 2 subsequent telephone calls to the users. If they still did not log in, staff would follow up with the user to encourage their participation [95].

By better understanding the components of motivation, promoters of healthy lifestyles can potentially design better interventions. Motivation is a likely explanation for the relationship between study adherence, intervention adherence, and behavioral outcomes. Intervention designers could potentially increase adherence by addressing the 2 common dimensions of motivation: participants’ goal-commitment and their ability/self-efficacy. For example, campaigns could benefit by intentionally designing online interventions around goals that appeal to the target audiences (following the social marketing approach), while also offering tailored support to aid participants who may lack ability or self-efficacy. Such an approach is similar to the Fogg behavioral model [93], which offers guidelines on when to address users’ motivation, ability, or both.

The capacity to develop mass-interpersonal online interventions may be limited by existing influence taxonomies that are not suitable to describing the psychological profile of interventions from an interpersonal or campaign perspective. During this study’s initial review of influence systems [35], no systems were identified that offered a full range of influence components within a theoretically based framework suitable to campaign applications. The CBICM developed for this meta-analysis [34,35] integrates influence research from various disciplines into a simple model that can aid intervention analysis or design whether interventions are modeled on interpersonal, mass-media, or mass-interpersonal interaction or whether they are modeled on one-way or two-way communication. However, the CBICM is only as good as the taxonomies it integrates. Within this study, the taxonomies of behavioral change techniques [38] and of behavioral determinants [50] proved to be highly robust coding instruments though they did not capture the full range of factors that may explain intervention efficacy. To compensate, it was necessary to add factors from persuasive technology and other behavioral science fields. During this meta-analysis, the CBICM proved to be an effective framework that can aid the science of online intervention research and design. Additionally, as a broad framework, there is scope to further expand and refine the CBICM.

### Limitations

The scope of online interventions in this study is limited to those targeting voluntary behavioral change, similar to the types of interventions conventionally used in social marketing campaigns for public health. While coding influence components, some papers only provided vague descriptions, while others did not describe influence components other than those that comprise conventional therapy. It would have been ideal to code influence components directly from the interventions rather than research papers. Control conditions were rarely described in enough detail to code relative influence components with full confidence. As some influence components were used more often than others, this study may offer more reliable figures for popular influence components, which draw from a larger pool of studies. As there are few studies of online interventions targeting voluntary behaviors, it was necessary to combine effect sizes across behavioral domains. It would have been ideal to have at least 2 coders from which intercoder reliability calculations could have been estimated.

Although authors of similar meta-analyses have conducted numerous univariate analyses to assess effect sizes associated with moderator variables [8,9], by calculating many influence component effect sizes, this approach may have led to type I errors: false positives. While there is consensus that numerous independent calculations will increase the odds of producing false positives, there is no consensus on how to handle this problem [56]. In light of this common methodological limitation, readers may reconsider the findings with a Bonferroni correction. The psychological analysis contained 52 independent univariate effect size calculations (excluding the demographic factors). Consequently, the CBICM presentation of influence component effect sizes may be judged in light of a Bonferroni correction where the traditional statistical significance test of less than .05 is divided by the number of independent effect size calculations (.05/52), which rounds up to a stringent significance test of less than .001. A sizable proportion of the psychological mediator analyses effect sizes met this conservative statistical significance test.

### Conclusions

The studies in this meta-analysis demonstrate that online interventions targeting voluntary behavior change can work. Compared with waitlists, they demonstrate moderate efficacy, while compared with print materials, they offer similar impacts but with the advantages of lower costs and broader reach.

In general, the interventions informed users about the consequences of their behavior, helped them set and achieve goals, taught them skills, and provided normative pressure. Feedback mechanisms were common, with many interventions using tailoring along with personalization and offering services to track and report users’ progress toward their goals.

Motivation may be the critical factor that drives study adherence, intervention adherence, and impact. Time proved to be a critical factor, with impacts and adherence appearing to fade over time, perhaps as motivation depreciated.

Psychological design appears relevant to intervention efficacy. Although the relationships between the number of influence components and behavioral outcomes were inconclusive, there may be a relationship: Too few influence components may not be enough to influence behavior, while too many may be counterproductive. However, there may be a middle ground comprising a modest number of relevant influence components.

These findings suggest it is feasible to deploy online interventions that target individual-level behavior change, which can be scaled to achieve population-level health benefits. Given the high-reach and low-cost of online technologies, the stage may be set for increased social marketing campaigns that blend mass-media outreach with interpersonal digital support. For example, this means fewer public health campaigns that just disseminate warnings or advice and more campaigns that offer online tailored support in the form of digital therapists that help citizens help themselves.

## Multimedia Appendix 1

Communication-based influence components model

## Abbreviations

ANOVA: analysis of variance
CBICM: communication-based influence components model
CI: confidence interval
