# Supplementary material for: Online Interventions for Social Marketing Health Behavior Change Campaigns: A Meta-Analysis of Psychological Architectures and Adherence Factors
Source: J Med Internet Res. 2011 Feb 14;13(1):e17. doi: 10.2196/jmir.1367 (PMC3221338; doi:10.2196/jmir.1367)
Supplement: Supplementary file 1 [file jmir_v13i1e17_app1.pdf]

Summary of the Communication-based Influence Components Model

Figure 1 presents the communication-based influence components model (CBICM) [1,2], a model that is used to integrate influence frameworks into a system suitable to analysing or designing online behavior change interventions.

There are a large number of influence frameworks that describe the factors that may influence the way people think and act. Health behaviour change literature, social marketing, therapy, persuasive communications, and evidence-based behavioural medicine all share common features. However, they differ greatly in their organizing principles, philosophies, and intended use. None are comprehensive enough to represent the full range of factors that may shape an intervention's efficacy.

To overcome this limitation, the CBICM provides a communication-based framework that can be used to integrate research from different fields into a simple and theoretically-based model. Once amalgamated, the model can be used to build a comprehensive checklist of factors that may influence intervention efficacy.

Figure 1: Communication-based Influence Components Model (CBICM)

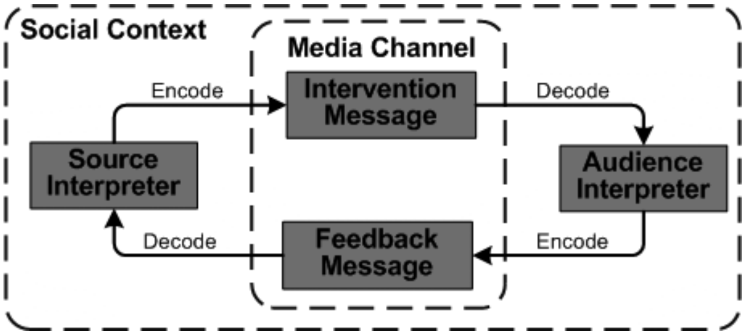

The CBICM is based on a circular communication model. Within each part of the communication process, there are a series of clusters that describe the social context, media channel, source, message, audience, and feedback. Each cluster of the CBICM contains factors that exert persuasive effects. The factors that can influence a person's psychology or behaviour are called influence components. The combination (summative, subtractive, interaction) of all influence components produces an intervention's overall effect.

For example, an intervention may comprise influence components form the source (credibility, likability, and similarity to the audience), the media channel (where video can excite emotions better than written words), and the intervention message (stressing the consequences of behaviour, skill building exercises, and behavioural monitoring). In each cluster, there are a range of influence components that can be combined, and which together, describe the psychological architecture of an intervention.

Table 1 presents the CBICM's clusters and describes them from the point of view of interaction between a therapists (source) and their client (audience).

This is the Multimedia Appendix to a full manuscript published in the Journal of Medical Internet Research:
Cugelman, B., Thelwall, M., & Dawes, P. Online interventions for social marketing health behavior change campaigns: A meta-analysis of psychological architectures and adherence factors. J Med Internet Res (2011). doi:10.2196/jmir.1367
http://dx.doi.org/10.2196/jmir.1367

Table 1: CBICM clusters

Cluster	Description
Social context	Environmental factors that can influence the client's behavor, such as the impact of family and friends, whether they increase or decrease the likelihood of achieving a goal
Media channel	The persuasive advantages associated with different communication vehicles, such as in-person, print, audio, video, or interactive media. Depending on the nature of the treatment, the therapist may prefer to use face-to-face meetings, phone calls, a workbook, or email
Source interpreter	Persuasive attributes ascribed to the therapist, such as their credibility, similarity to the client, physical appearance, or likeability
Source encoding	The impact of how the therapists express the treatment to their client, such as whether they encode it as a single or multiple sessions, or the tone of language they use to express their therapy
Intervention message	The specific therapy or treatment offered to the client
Audience interpreter	The psychological faculties targeted by an intervention, which include constructs used by common behaviour change theories. For example, the therapist may focus on the client's beliefs about the consequences of their behavior, self efficacy, and also make social norm appeals
Audience encoding	How the client encodes their feedback to the therapist such speaking in person, communicating by a web interface, or though data download from a monitoring device. Some feedback mechanisms may be less demanding than others, prompting the therapist to invite their patient to encode their feedback in the most convenient format
Feedback message	The persuasive techniques that can only be employed after receiving feedback, which are integrated into an intervention. By receiving feedback from the patient, the therapist can personalize and tailor the therapy to their client's particular preferences and needs. However, in one-way interventions, the CBICM omits feedback clusters, rendering these techniques impossible to employ

The CBICM is flexible and can be used to describe a wide diversity of interventions. As it is based on a circular model, it can describe different communication approaches, whether they are based on traditional one-way marketing, or interactive two-way relationship building. It can describe interventions at an individual level, such as between a therapist and client, or at a population level, such as between an organization and their constituents. Depending on the intervention source, the CBICM can describe persuasive communication effects, whether they are conveyed from a person to another person, from a publication to a person, or from interactive technology to its user.

Although the model was developed for online interventions, it also has application to traditional interventions, as well as interactive engagement, such as social media campaigns. The CBICM may be used as a research tool to aid the analysis of existing interventions or as a tool to aid development online behavior change interventions. For a detailed description, refer to [1,2].

References

[1] Cugelman B, Thelwall M, Dawes P. Communication-based influence components model. in Persuasive. Claremont: ACM 2009

[2] Cugelman B. Online social marketing: Website factors in behavioural change, in School of Computing and IT, University of Wolverhampton: Wolverhampton. 2010

This is the Multimedia Appendix to a full manuscript published in the Journal of Medical Internet Research: Cugelman, B., Thelwall, M., & Dawes, P. Online interventions for social marketing health behavior change campaigns: A meta-analysis of psychological architectures and adherence factors. J Med Internet Res (2011). doi:10.2196/jmir.1367 http://dx.doi.org/10.2196/jmir.1367
